# Therapist use of cognitive behavior therapy and eye movement desensitization and reprocessing components for the treatment of posttraumatic stress disorder in practice settings

**DOI:** 10.3389/fpsyg.2023.1158344

**Published:** 2023-10-19

**Authors:** Erin L. Neill, Amie Zarling, Carl F. Weems

**Affiliations:** Human Development and Family Studies, Iowa State University, Ames, IA, United States

**Keywords:** traumatic, PTSD, implementation, CBT, EMDR, translation

## Abstract

**Introduction:**

Treatment practice guidelines for posttraumatic stress disorder (PTSD) recommend both Eye Movement Desensitization and Reprocessing (EMDR) and Cognitive Behavior Therapy (CBT); however, implementation in practice setting remains challenging. Here we aim to foster implementation efforts for PTSD by identifying the relative use of the various components of empirically supported treatments by therapists and the characteristics that predict their use.

**Methods:**

Surveyed 346 therapists (84.07% female) of whom 272 participants (78.61%) were trained primarily in CBT and 135 participants (39.02%) were trained in primarily in EMDR. Assessed relative use of various EMDR and CBT components as well as several training and personality factors.

**Results:**

Psychoeducation about trauma was the most common element used. “Off label” use of components was also identified with application of EMDR techniques to other diagnoses. Findings also suggest underutilization of *in vivo* exposure techniques across therapists. EMDR therapists reported relatively high use of core EMDR techniques (i.e., greater use of EMDR core techniques). Big five personality factors, therapy efficacy, and anxiety were associated with differential component use.

**Discussion:**

Results identify trends in empirically supported component use and therapist characteristics that are associated with the use of various techniques for PTSD. The findings suggest implementation efforts could foster training in underused techniques, address barriers to their utilization and develop knowledge of effective packages of components.

## Introduction

Exposure to extreme adversity traumatic events can lead to life-disrupting consequences including poor school performance, behavioral problems, strains on interpersonal relationships, job loss or prolonged unemployment, lower income, a higher risk for suicidal ideation and attempts, substance use, and mental health disorders such as depression, anxiety, and posttraumatic stress disorder (PTSD) (Bradley et al., 2005; Cohen et al., 2006). Fortunately, resilience to the negative effects can be fostered as treatments for PTSD are efficacious. For example, both CBT and EMDR have been recommended for the treatment of PTSD (ISTSS, 2018). While CBT and EMDR are empirically supported treatments (EST) that can ameliorate distress and foster resilience, their implementation in community and other practice settings remains a challenge (Herschell et al., 2010; Weems, 2019). Many studies have found that the parameters of a *clinical trial* for an EST vary substantially from *real-world practice*, and so it is not surprising that clients in community trials have tended to have worse outcomes than those in clinical trials (Weisz et al., 2015).

Exposure techniques are one of the most empirically supported components of intervention for PTSD but may be some of the most under-used (Farrell et al., 2013; Harned et al., 2013). Researchers and clinicians cite many reasons for this, including potential iatrogenic effects of *in vivo* exposure, clients being unable to tolerate exposure, and other ethical considerations (Farrell et al., 2013). Although most CBT manuals for PTSD include exposure sessions, and exposure is an EST for PTSD, research has shown that often therapists outside of a research environment are not using exposure sessions in their practice (Foy et al., 1996; Becker et al., 2004; Cook et al., 2004; Borntrager et al., 2013).

Characteristics of individual therapists such as their counseling efficacy may influence their selection, use, and evaluation of EST’s (Larson et al., 1992). Larson et al. (1992) applied self-efficacy theory and principles to counselors, creating the Counseling Self-Estimate Inventory (COSE). In addition to counseling self-efficacy, anxiety and personality characteristics (such as those from the Big five openness to experience, conscientiousness, extraversion, agreeableness, and neuroticism, Caprara, 2001) may also predict EST component use. Larson et al. (1992) found that both state and trait anxiety (as measured by the STAI) were significantly negatively related to the total and five factor scores on the COSE. That is, those therapists with higher feelings of counseling self-efficacy had lower feelings of anxiety, and neuroticism. However, as noted above there is likely to be differential use of various components of ESTs, such as exposure, which may be differentially associated with personality factors or the type of EST training one received.

Chorpita et al. (2005) proposed the “Distillation and Matching Model” as a way to empirically factor intervention literature to come up with “profiles” from evidence-based approaches for use in community practice. These profiles are useful because they can then be matched to clients in community practice based on which problems they want to target, or even the client’s demographic factors such as age, gender, or ethnicity (Chorpita et al., 2005). This model aims to solve several of the current problems in the treatment literature including helping clinicians understand how treatments may be similar and different, guide treatment selection based on client characteristics or needs, as well as possibly create new interventions in the future (Chorpita et al., 2005). Applied, the distillation and matching model can provide a map for clinicians to find the treatment components with evidence for the most favorable treatment outcome, or the components best catered to their client’s individual differences (Chorpita and Daleiden, 2009). For traumatic stress, they found that exposure (0.91), cognitive elements (0.91), psychoeducation for the child (0.82), followed by relaxation (0.64), psychoeducation for the parent (0.45), and maintenance/relapse prevention (0.45) were the most common practice elements in the 11 treatments examined (Chorpita and Daleiden, 2009).

The question remains, what are clinicians in practice settings actually doing in their practice? Are they using empirically supported treatments, just some components of those treatments, or are they using any treatment manuals? Garland et al. (2010) suggested that the place to start is to find out exactly what is happening in “usual care” in practice settings. This topic has received growing attention over the years (Garland et al., 2010) with a meta-analysis of 32 studies that directly compared EST’s to usual care by Weisz et al. (2006) finding that EST’s were superior in treating mental illness. Worryingly, one study found that if youth had PTSD as a diagnosis, they were significantly less likely to receive an EST (Borntrager et al., 2013).

The purpose of this study was to examine the use of individual components of CBT and EMDR that are being used by therapists in community practice. First, this research aims to add descriptive information about the use of various EST techniques and about therapists’ use of the components of CBT and EMDR. Next, we theorized that the components would load onto at least two factors: one with mainly CBT techniques and one with EMDR techniques (there may also be a factor with items crossing the two techniques). Given that at least two factors emerge, one with unique CBT and one with unique EMDR components (additional cross-loading factors are also expected to emerge) we hypothesized that exposure would be relatively infrequently used. We also predicted that EMDR therapists will use EMDR techniques at a higher frequency than CBT therapists will use CBT techniques. The theoretical reasons for this hypothesis include (a) the knowledge that CBT with exposure sessions has the most empirical evidence for the treatment of PTSD but is also an underutilized treatment (Farrell et al., 2013; Harned et al., 2013); (b) there is one main, generally agreed upon treatment protocol for EMDR and any trainings have to follow the EMDR protocol; since it is uniform, anyone trained in EMDR will be trained not only the same way, but also taught to follow the entirety of the manual or treatment protocol. Finally, based on the theoretical predictions and empirical evidence to date outlined above we predicted clinicians who rate higher on the “Big Five” personality traits extraversion, openness to experience, and agreeableness, as well as those with lower levels of anxiety and higher ratings of counseling self-efficacy, will be more likely to use the broad range of therapeutic components found in CBT and EMDR manuals.

## Materials and methods

### Participants

Participants were recruited by using IRB approved study scripts and flyers through the following methods: electronically contacted therapists known through networking with other therapists, listserv and online bulletin board administrators for professional organizations, social media sites such as Facebook, to post in relevant online groups, bought access to a mailing list for marriage and family therapists (email addresses were not available) and mailed a study flyer to a random sample of 2,000 marriage and family therapists. See Supplementary material A for information about all organizations contacted and whether, and how, they distributed information about the study to recruit participants as well as how many participants were licensed in each state and additional demographic information. Briefly, four hundred sixty-two potential participants followed a link to an online survey and agreed to the informed consent by clicking that they agreed to participate in the study. Of those 434 participants who qualified for the study, 425 participants (97.93%) answered that they see clients who have experienced traumatic events. Although those participants who did not see clients who had experienced traumatic events were allowed to continue the survey, all nine participants who answered that they did not see trauma in their practice also chose not to answer the next questions on the survey – the *Therapists’ Experiences with Empirically Supported Treatments Questionnaire* (EST-Q) developed for this study (see below). Finally, participants who did not complete at least 80% of the EST-Q items (at least 26 of the 32 items) were not included in the current study. There were 116 participants (25.11%) who were excluded because they completed 26 or fewer EST-Q items (76 potential participants, or 16.45% of the total potential participants, did not complete even one question on the EST-Q). Inclusion criteria for this study included, (1) being a licensed therapist (social worker, psychologist, marriage and family therapist, mental health counselor, etc.) who, (2) spends at least 50% of their professional time seeing clients for individual therapy. There was not a requirement for therapists to see clients who have experienced trauma and PTSD because of the desire for a broader sample (therapists use the empirically-supported treatment CBT for depression, anxiety, etc.), as well as research (e.g., Liu et al., 2017) finding that most people experience some type of trauma in their lives. Participants that practice in a community setting were the focus of recruitment; however, participants were not excluded based on their practice setting. Those therapists still in training (graduate school) were excluded from this study (those who are licensed in their profession would not be in graduate school). Previous research in the field has focused on convenience samples of graduate students in training, but the focus of the current study aimed to expand from that and recruit participants who have completed their training in order to understand what is currently happening in community therapists’ offices.

The final sample of 346 participants was primarily female (285 participants; 84.07%) and white (294 participants; 86.98%). Participants ranged in age from 24 to 80 years old, with the mean age being 44.59 years. On average, the participants in the current study had completed their graduate degree 13.26 years ago and saw 21 clients per week. This sample is representative of mental health professions such as social work where the vast majority of practitioners are female and white (Salsberg et al., 2017). See Supplementary material A for more complete participant demographic information.

### Measures

As noted above, the EST-Q was designed specifically for this study and is the main outcome measure for the current study. While previous similar measures exist, they either do not have EMDR items (Hogue et al., 2014) or ask about deviations from protocol versus actual content (Lau et al., 2017). Specifically, it combines essential elements of CBT and EMDR. All the items are available in Table 1 and in Supplementary material B and asks, “Thinking only about your clients who are in treatment for PTSD symptoms or trauma exposure, please consider the components of Empirically-Supported Treatments listed below. Please check how often in your practice you use each component with survivors of trauma.” The scale for the EST-Q is a 5-point Likert scale ranging from “1 = Never (I never use this with any of my clients)” to “5 = Always (I always use this with all of my clients).” Items specific to EMDR were taken from the EMDR protocol (Shapiro, 2001) and the EMDR Humanitarian Assistance Program manual. Items specific to CBT were taken from the CBT protocols (e.g., Cohen et al., 2006) and the CBT components identified in Chorpita and Daleiden (2009). Supplementary Table B in the supplement breaks down items by CBT versus EMDR. Mean scores on the EST-Q items were used in this study. The possible scores for the total EST-Q therefore range from 1–5. Additional psychometric data are presented in the results section.

**Table 1:** Frequency of EST-Q items – essential EMDR and CBT elements presented to therapists.

			Frequency				
	*M* (*SD*)	*n*	Never	Rarely	Sometimes	Often	Always
Psychoeducation – provide the client information about traumatic experiences, trauma reactions, symptoms, and trauma reminders	4.62 (0.68)	363	0	8	18	77	260
Work on emotion knowledge/affect identification and emotion regulation/modulation skills	4.43 (0.76)	358	2	7	26	123	200
Explain that processing of trauma memories may continue after the session	4.41 (0.87)	357	5	11	29	98	214
Deep breathing exercises or breathing training	4.36 (0.82)	363	3	9	35	122	194
Increase awareness of problem-solving skills and/or social skills	4.25 (0.83)	355	1	11	51	129	163
Reevaluation – Check to make sure the client’s positive results have been maintained	4.24 (0.91)	357	7	10	44	125	171
Address personal safety skills and assertive communication	4.23 (0.85)	358	4	8	48	141	157
Use of cognitive restructuring with client (thought-feeling model, connect negative feelings to thoughts, challenge thoughts, generate alternative thought, practice alternative thoughts)	4.00 (0.96)	354	7	15	76	129	127
Help client establish a calm/safe place in their mind to “go” when traumatic memories are too much	3.95 (1.11)	363	18	22	58	128	137
Review with client previous homework – praise efforts and troubleshoot obstacles	3.94 (1.02)	363	14	18	61	154	116
Use of guided imagery/imaginal exposure	3.88 (1.04)	362	15	23	62	154	108
Utilize homework and other educational materials – informational handouts, worksheets, etc. with client	3.82 (1.01)	362	8	29	85	137	103
Have client do body scan (i.e., “Where do you feel the trauma in your body?”)	3.76 (1.11)	358	19	28	76	131	104
Provide progressive muscle relaxation (or provide other progressive relaxation skills)	3.71 (0.97)	359	10	29	86	163	71
Use of homework assigning (e.g., develop homework assignment, collaborate with client, make specific plan, troubleshoot obstacles)	3.70 (1.03)	355	10	35	91	135	84
Agenda setting – articulate & implement a specific agenda for session, identify other issues	3.64 (1.08)	369	18	32	100	133	86
Identify processing targets from positive and negative events in client’s life (i.e., first or worst traumatic event)	3.61 (1.21)	368	36	25	79	135	93
Establish a stop signal for when traumatic memories are too much to continue processing/end of session	3.60 (1.32)	363	37	42	66	101	117
Elicit image of the traumatic event, negative belief currently held, desired positive belief, current emotion(s), and physical sensation (body location)	3.60 (1.20)	358	27	39	77	121	94
Help client develop a trauma narrative	3.55 (1.08)	352	21	28	109	126	68
Use of Subjective Units of Disturbance Scale (“SUDS”) “How disturbing does it feel to you now?”	3.38 (1.43)	354	61	37	63	92	101
Assign thought record or daily diary to client (Client to record thoughts, feelings/emotions, behaviors/actions)	3.04 (1.02)	352	23	83	126	96	24
Have the client imagine a container to hold memories/thoughts when not working through them	3.00 (1.39)	369	79	56	82	90	62
Use of Validity of Positive Cognition (“VOC”) “How true do those words ____feel to you now?”	2.89 (1.47)	354	95	54	63	78	64
Use of a standard measure prior to session to assess client’s level of symptoms for the day’s session	2.83 (1.32)	368	75	84	85	76	48
I work with my clients to create a graded exposure hierarchy.	2.77 (1.27)	358	83	60	103	81	31
Provide client an explanation of Eye Movement Desensitization and Reprocessing	2.70 (1.69)	362	156	31	31	55	89
Use “Cognitive Interweave” to open blocked processing by elicitation of more adaptive information	2.54 (1.48)	354	144	30	66	72	42
Bilateral stimulation with negative cognition and traumatic event (e.g., eye movements, tactile or visual stimulation, etc.)	2.45 (1.61)	355	180	16	30	76	53
I always work through the entire graded exposure hierarchy.	2.43 (1.16)	358	100	87	102	55	14
Bilateral stimulation with positive cognition (e.g., eye movements, tactile or visual stimulation, etc.)	2.40 (1.58)	354	182	18	33	73	48
Use of *in-vivo* exposure	2.31 (1.19)	359	121	88	82	54	14

The Big Five (John and Srivastava, 1999) personality traits of openness, conscientiousness, extraversion, agreeableness, and neuroticism were assessed using the BFI (John et al., 1991, 2008). Respondents are presented with 44 characteristics (e.g., “Is original, comes up with new ideas”) and asked to rate their personal applicability along a five-point Likert scale (1 = “Disagree Strongly,” 5 = “Agree Strongly”). Subscale scores are created by averaging responses to items representative of each personality trait. In the current study, the subscales range in internal consistency: Neuroticism (*α* = 0.83), Agreeableness (*α* = 0.73), Extraversion (*α* = 0.85), Conscientiousness (*α* = 0.83), and Openness (*α* = 0.79).

The Counseling Self-Estimate Inventory (COSE; Larson et al., 1992) is a 37-item instrument created to measure counseling self-efficacy, or how mental health therapists feel about their own skills. The randomly ordered statements include both positive and negative wording that ask participants to rate themselves on how they feel they would actually perform a counseling skill during a counseling interview at the present time. The COSE uses a six-point Likert scale from 1 – *strongly disagree* to 6 – *strongly agree*. Higher scores on the COSE reflect stronger perceptions of the therapists’ self-efficacy. Larson et al. (1992) found that the total instrument had good internal consistency (*α* = 0.93). They also found that there were five factors with a range of internal consistency: Microskills (*α* = 0.88), Process (*α* = 0.87), Difficult Client Behaviors (*α* = 0.80), Cultural Competence (*α* = 0.78), and Awareness of Values (*α* = 0.62). Reliability estimates for the current study include: Total COSE (*α* = 0.89), Microskills (*α* = 0.84), Process (*α* = 0.81), Difficult Client Behaviors (*α* = 0.70), Cultural Competence (*α* = 0.74), and Awareness of Values (*α* = 0.27).

The *State–Trait Anxiety Inventory – Form Y* (STAI; Spielberger et al., 1983) is a 20-item self-report measure of anxiety symptoms. The STAI measures both state (temporary condition in specific situations) and trait (general tendency to perceive situations as threatening) anxiety (Spielberger, 1966). It indicates the intensity of anxious feelings and was chosen for this study because it was initially developed as a measure to study anxiety in normative adult populations. Items include statements such as, “I feel at ease” (state) or “I am a steady person” (trait). They are rated on a 4-point Likert scale from “1 = not at all” to “4 = very much so.” STAI items have demonstrated good test–retest reliability (*r* = 0.69–0.86), as well as an acceptable level of internal consistency across samples (*α*’s > 0.80) (Spielberger, 1966). In the current study, internal consistency was excellent with trait anxiety reliability (*α* = 0.92) and state anxiety reliability (*α* = 0.93).

### Procedures

The University Office for Responsible Research’s Institutional Review Board reviewed and approved this study (IRB #17–204). Participants were recruited for a survey study on therapists’ experiences with empirically-supported treatments in an online format. The participants were given a Qualtrics link (either from an electronic or paper study flyer, or through email distribution) where they first read and completed informed consent and then proceeded onto the questionnaires. Contact information for the investigator was available if participants had any questions or concerns. The survey also asked basic demographic questions, training and therapist experiences. Those who were trained in CBT indicated that they were trained in a variety of ways including certificate programs, continuing education, and many were trained in graduate school. Training in EMDR again ranged from completing an EMDR HAP course, continuing education courses or certificate programs, training with the EMDR Institute or a certified institute trainer, and three participants answered that they were trained in graduate school. Further questions in the survey asked open-ended questions, such as, “Are there any problems/disorders for which you feel using CBT/EMDR is inappropriate?” Additional details are provided in the results section.

### Data analyses

The data for this cross sectional correlational survey was initially analyzed using descriptive and inferential statistics using SPSS software (SPSS, 2012). Exploratory factor analysis was be performed on the EST-Q items with the identified scales’ internal consistency assessed with coefficient alpha. Differences across therapist type was tested with ANOVA models and correlations among personality traits self-efficacy, anxiety, and use of empirically supported treatment (CBT and EMDR) components were examined with person correlations and regression analysis.

## Results

### Descriptive statistics use of the components of CBT and EMDR

Participants were asked to rate themselves with the following statement, “I follow a treatment manual closely,” as “very true,” “somewhat true,” or “not true.” Of the 325 participants who answered the question, only 53 participants (16.31%) felt the statement was “very true,” while 144 (44.31%) felt it was “somewhat true,” and 128 (39.38%) felt it was “not true” for them. Participants were also asked whether they use a structured questionnaire to diagnose PTSD in a client. A slight majority of the 323 participants who answered the question (174 participants; 53.87%) answered that they did not use a structured questionnaire to diagnose PTSD in a therapists client, while 149 participants (46.13%) said that they did use a structured questionnaire.

Table 1 presents the average frequency therapists indicated as to how often they use each of the 32 different elements of CBT and EMDR assessed on the EST-Q (this frequency table is also broken down by type of CBT versus therapist in Supplementary material B, Supplementary Tables B1, B2). Two hundred seventy-two participants (78.61%) answered that they were trained in Cognitive Behavioral Therapy, or CBT. Of those trained in CBT, all but 16 participants (256 participants; 94.12%) – stated that they use CBT in their practice. When asked what types of problems they treat using CBT, 242 participants (88.97%) selected “anxiety disorders,” 236 participants (86.76%) selected “depression,” 215 participants (79.04%) selected “PTSD/trauma exposure,” 134 participants (49.26%) selected “marital/relationship problems,” 113 participants (41.54%) selected “addictions,” 89 participants (32.72%) selected “health problems,” 68 participants (25%) selected “eating disorders,” and 42 participants (15.44%) selected “other.” Those who selected “other” indicated they also used CBT to treat ADHD (*n* = 5), behavior problems or disorders (*n* = 4), bipolar disorder (*n* = 3), chronic pain or health problems (*n* = 3), obsessive-compulsive disorder (*n* = 4), anger problems or anger management (*n* = 2), schizophrenia or other serious mental illness (*n* = 4), borderline personality disorder and other personality disorders (*n* = 4), sex therapy (*n* = 1), sex offending or sexual compulsions (*n* = 3), autism (*n* = 1), attachment (*n* = 1), and psychosis (*n* = 1) among a few other disorders listed.

Participants were also asked, “are there any problems/disorders for which you feel using CBT is inappropriate?” Interestingly, the most common response regarding problems/disorders for which CBT is inappropriate was that CBT was not appropriate for treating trauma or PTSD (*n* = 31). Other common responses of problems or disorders therapist participants felt that it was inappropriate to use CBT to treat included anxiety (*n* = 4), attachment (*n* = 6), personality disorders and in particular borderline personality disorder (*n* = 8), interpersonal problems (*n* = 4), couples and/or family therapy (*n* = 11), those with a cognitive disability or low cognitive functioning or IQ (*n* = 13), those who “intellectualize” or with a high IQ (*n* = 6), grief (*n* = 5), dissociative clients (*n* = 6), those with active psychosis (*n* = 5), young children (*n* = 9), and those who have not previously benefitted from CBT (*n* = 8).

One-hundred-thirty-five participants (39.02%) who completed the survey answered that they were trained in EMDR. Of those trained in EMDR, all but four participants (131 participants; 97.04%) stated that they use EMDR in their practice. When asked what types of problems they treat using EMDR, 130 participants (96.30%) selected “PTSD/trauma exposure,” 109 participants (80.74%) selected “anxiety disorders,” 94 participants (69.63%) selected “depression,” 53 participants (39.26%) selected “addictions,” 50 participants (37.04%) selected “marital/relationship problems,” 42 participants (31.11%) selected “health problems,” 35 participants (25.93%) selected “eating disorders,” and 22 participants (16.30%) selected “other.” Those who selected “other” indicated they also used EMDR to treat ADHD, attachment (*n* = 3), children’s behavior issues, chronic pain (*n* = 2), dissociation (*n* = 3), grief or loss (*n* = 3), fostering or adoption, obsessions and/or compulsions (*n* = 2), panic attacks, secondary trauma or burnout, self-esteem issues, separation anxiety, sex therapy.

Participants were also asked, “Are there any problems/disorders for which you feel using EMDR is inappropriate?” The most common response (*n* = 15) was that clinicians would not use EMDR to treat clients who were dissociative or who had a dissociative disorder. Ten therapists stated that they would not use EMDR to treat someone with active psychosis while six therapists stated they would not use the modality for those clients actively using substances. Eleven therapists stated that there were “many” disorders for which they would not use EMDR for treatment, with one saying there were “too many to list.” Multiple therapists also listed other specific problems/disorders they would not use EMDR to treat, such as borderline personality disorder and other personality disorders (*n* = 6), those in couples or family therapy (*n* = 4), grief (*n* = 3), a pregnant client (*n* = 3), adjustment disorders (*n* = 2), attention disorders or ADHD (*n* = 2), those who had an upcoming court case (*n* = 2), schizophrenia (*n* = 2), those with suicidal ideation (*n* = 2), and two therapists stated they would not use EMDR with children.

### Hypothesis that the components would load onto at least two factors

The factor structure of the items on the EST-Q was examined using exploratory factor analysis. Additional details including factor loadings are in Supplementary material B (Supplementary Table B3 and Supplementary description). A four-factor model appeared to be the best fit and accounted for 55.26% of the total variance. The four subscales that emerged all had good internal consistency (> 0.75) and were named CBT (*α* = 0.80), EMDR (*α* = 0.93), Both (*α* = 0.77; a factor containing items that are representative of both CBT and EMDR modalities), and exposure (*α* = 0.81; a factor for items relating to exposure techniques). Factor loadings are available in the Supplementary material B, 13 items loaded onto the uniquely “EMDR” factor, seven items loaded onto the uniquely “CBT” factor, eight items loaded onto a “both” CBT and EMDR factor, and four items loaded onto an “exposure” factor. Examination of the salient loadings (see Supplementary material B) suggested that the four factors were consistent with expectations regarding the loading of the items onto EMDR and CBT related component factors. That is, the data from the EST-Q measure loaded onto a unique CBT factor and a unique EMDR technique factor. Additionally, at least two items, psychoeducation about trauma and progressive muscle relaxation, were expected to cross-load onto both factors, as these two components are part of both CBT and EMDR – these items and six other items loaded onto a third factor that appears to have elements common to both CBT and EMDR. Finally, a fourth factor, “exposure,” also emerged with four items and is theoretically consistent with previous research identifying a small subset of therapists who are trained in or willing to use exposure therapy to treat trauma (Farrell et al., 2013; Harned et al., 2013).

### Hypothesis that exposure would be relatively infrequent and that EMDR therapists will use EMDR techniques at a higher frequency than CBT therapists

A four by four (therapist type [4] by EST-Q subscale [4]) mixed effects repeated-measures factorial ANOVA was conducted with a significant therapist type by subscale interaction anticipated. Scores for each subscale of the EST-Q were derived by taking the mean of all subscale items, placing overall subscale score along the same 0–4 point range used in the measure, and providing a clear metric for interpretation (e.g., a score of 2 on the EMDR subscale indicates the participant ‘Sometimes’ uses EMDR components). Type of therapist (EMDR, CBT, both, or none) was defined by therapists’ answers to two questions in the survey (1) “Are you trained in Cognitive Behavioral Therapy, or CBT?” and (2) “Are you trained in Eye Movement Desensitization and Reprocessing, or EMDR?” Each of these questions allowed therapist participants to check either “yes” or “no” to answer the question. One-hundred-sixty-two participants responded that they were trained in CBT only, 30 participants responded they were trained in EMDR only, 102 participants responded that they were trained in both CBT and EMDR, and 22 participants responded that they were trained in neither CBT nor EMDR.

**Figure 1:**
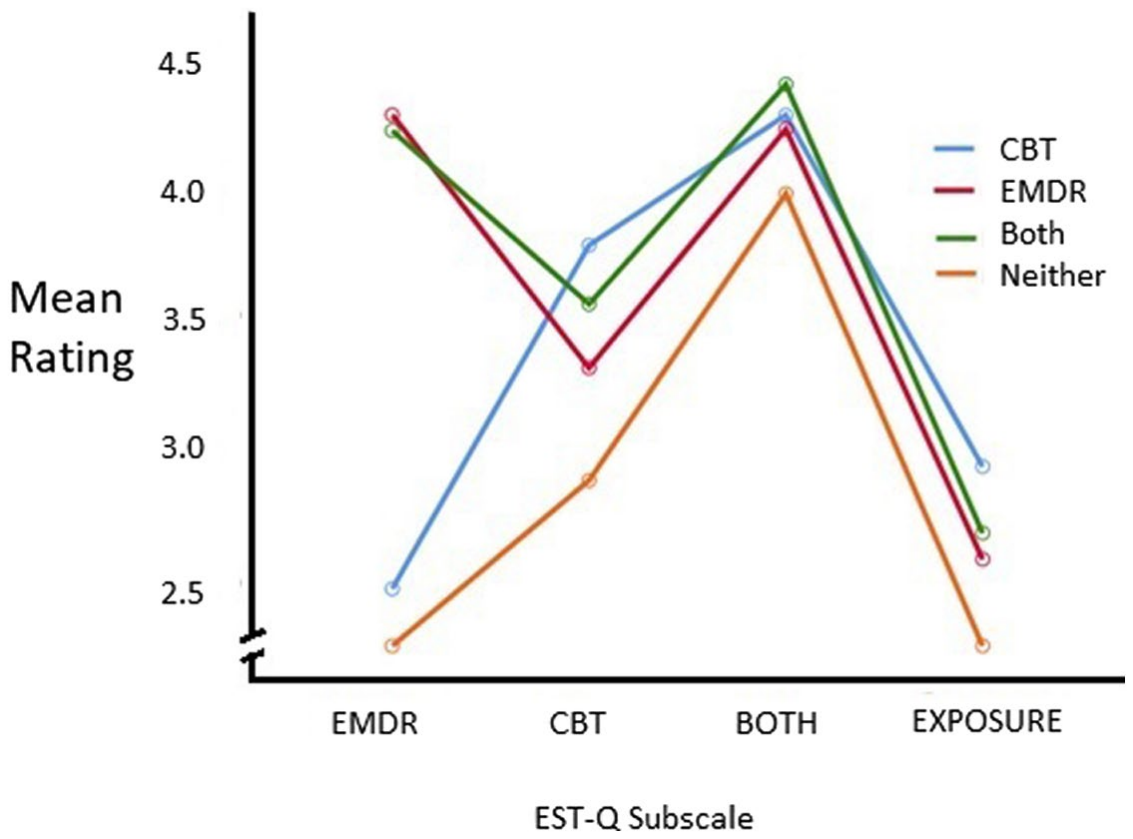
Means across subscales for each type of therapist. Type of therapist (EMDR, CBT, both, or none) was defined by therapists’ answers to two questions in the survey (1) “Are you trained in Cognitive Behavioral Therapy, or CBT?” and (2) “Are you trained in Eye Movement Desensitization and Reprocessing, or EMDR?”.

Figure 1 depicts the means across the subscales for type of therapist (n’s, means and standard deviations across are detailed in Supplementary Table B4). Mauchly’s test indicated that the assumption of sphericity had been violated, (χ^2^ (5) = 111.50, *p* < 0.001), therefore degrees of freedom were corrected using Huynh-Feldt (*ε* = 0.82) (Field, 2013). The results show that there was a significant main effect of the EST-Q subscales, [*F*(2.47, 786.27) = 220.00, *p* < 0.001] a significant between-subjects effect of therapist type [*F*(3, 319) = 21.82, *p* < 0.001] and also a significant interaction between the type of therapist and EST-Q subscale *F*(7.39, 786.27) = 72.82, *p* < 0.001. The interaction effect suggests that the profile of ratings across different types of therapists was different for different EST-Q subscales generally consistent with the hypothesis and therefore main effects should be interpreted but understood in the context of the interaction. The interaction was decomposed with a series of follow up analyses. There were significant differences across therapist types on each of the subscales EMDR subscale [*F*(3, 322) = 200.95, *p* < 0.001], CBT subscale [*F*(3, 320) = 15.04, *p* < 0.001], ‘Both’ subscale [*F*(3, 322) = 5.57, *p* = 0.001], and Exposure subscale [*F*(3, 321) = 4.73, *p* = 0.003]. The nature of these differences in subscale score was examined using a series of post-hoc tests contrasting scores across therapist types. Testing was performed using the Games-Howell procedure which accounts for differences in variances stemming from imbalanced group sizes (Field, 2013). Indeed, Levene’s test was significant for two of the EST-Q subscales (EMDR and Both), reflecting heterogenous variance in subscale scores across therapist-types, and further indicating that the use of the Games-Howell procedure was appropriate (Field, 2013).

The full results of the *post hoc* tests of between group differences on EST-Q subscale scores are presented in Supplementary material B (Supplementary Table B5). Main contrasts of interested are summarized here. As predicted, EMDR therapists scored significantly higher than CBT (*p* < 0.001) or “Neither” (*p* < 0.001) therapists on the EMDR subscale, but not therapists cross-trained in CBT and EMDR (‘Both’; *p* = 0.97). Moreover, “Both” therapists tended to show higher scores on the EMDR subscale than CBT therapists and “Neither” therapists (both *p* < 0.001). Regarding the CBT subscale of the EST-Q, as predicted CBT therapists reported significantly higher scores than all other type of therapists – EMDR (*p* < 0.01), “Both” (*p* = 0.05), “Neither” (*p* < 0.001). Additionally, “Both” therapists scored higher on the CBT subscale than “Neither” therapists (*p* < 0.01). There were no statistically significant differences between the types of therapists on the Both EST-Q subscale. Analyses with the Exposure subscale revealed a higher score in CBT therapists in contrast to ‘Neither’ therapists (*p* < 0.01). Additional follow up tests also showed that EMDR therapists did in fact have higher mean scores on their EMDR subscale than CBT therapists had on their CBT subscale [*t*(194) = 3.937, *p* < 0.001]; therapists who reported being trained in “Both” CBT and EMDR, had significantly higher mean scores on the EMDR subscale than the CBT subscale [*t*(208) = 6.583, *p* < 0.001], indicating that therapists trained in both CBT and EMDR were more likely to endorse using more EMDR treatment elements more often than CBT treatment elements.

### Hypotheses predicting component use by therapist characteristics

Means, standard deviations, and correlations for all study variables are presented in Table 2. As shown in Table 2, agreeableness was significantly and positively related to the subscales of EMDR and both. Agreeableness was also significantly and negatively associated with the exposure subscale, but not significantly related to the EST-Q CBT scale. Openness to experience was significantly, and negatively, related to the exposure scale of the EST-Q indicating that therapist participants who rated higher on openness to experience personality characteristics also were significantly less likely to use exposure elements. Supplemental regression analyses predicting EST-Q subscales while controlling for demographics and other therapist characteristics were conducted. Each EST-Q subscale (EMDR, CBT, Both, and Exposure) were each entered separately as the dependent variable (for four separate analyses), and personality traits of extraversion, agreeableness, openness to experience, conscientiousness, and neuroticism, as well as self-efficacy subscales, state and trait anxiety, and several other covariates including type of therapist training – (the four types CBT, EMDR, Both, or Neither were dummy coded EMDR versus other, Both versus other and neither versus other), age, gender, years practicing, type of degree – (masters or doctoral, and years since completing their terminal degree) were entered simultaneously as independent variables to predict each of the four subscales separately. The big 5 personality indicators were not predictive of any EST subscale but the counseling self-efficacy difficult client behaviors subscale remained significantly associated with the EST-Q EMDR, CBT and Both Subscales. Details are presented in Supplementary material C.

**Table 2:** Means, standard deviations, and correlations among study variables.

	Mean (SD)	*n*	1	2	3	4	5	6	7	8	9	10	11	12	13	14	15
1. Openness	39.41 (5.88)	289	-														
2. Conscientiousness	37.54 (5.63)	289	0.03	-													
3. Extraversion	27.96 (6.37)	291	0.29^***^	0.13^*^	-												
4. Agreeableness	38.54 (4.39)	289	0.22^***^	0.28^***^	0.18^**^	-											
5. Neuroticism	19.24 (6.03)	289	−0.29^***^	−0.40^***^	−0.35^***^	−0.49^***^	-										
6. Micro-skills	62.56 (6.22)	253	0.17^**^	0.39^***^	0.22^***^	0.42^***^	−0.43^***^	-									
7. Process	48.51 (8.19)	252	0.16^*^	0.35^***^	0.20^**^	0.28^***^	−0.41^***^	0.50^***^	-								
8. Difficult Behaviors	33.64 (5.00)	259	0.14^*^	0.31^***^	0.23^***^	0.28^***^	−0.38^***^	0.40^***^	0.59^***^	-							
9. Cultural Competence	20.05 (3.15)	250	0.15^*^	0.25^***^	0.16^**^	0.30^***^	−0.33^***^	0.45^***^	0.44^***^	0.36^***^	-						
10. Awareness	19.36 (2.83)	250	0.09	0.32^***^	0.06	0.33^***^	−0.25^***^	0.32^***^	0.39^***^	0.25^***^	0.40^***^	-					
11. State Anxiety	31.32 (8.92)	289	−0.22^***^	−0.42^***^	−0.23^***^	−0.39^***^	0.65^***^	−0.39^***^	−0.37^***^	−0.36^***^	−0.25^***^	−0.18^**^	-				
12. Trait Anxiety	33.78 (8.63)	286	−0.25^***^	−0.51^***^	−0.31^***^	−0.41^***^	0.79^***^	−0.42^***^	−0.41^***^	−0.37^***^	−0.36^***^	−0.26^***^	0.75^***^	-			
13. EMDR	3.17 (1.05)	346	0.12	0.13^*^	0.06	0.15^*^	−0.21^***^	0.19^**^	0.10	0.20^**^	0.25^***^	0.20^**^	−0.09	−0.20^**^			
14. CBT	3.58 (0.71)	344	−0.10	0.07	0.11	0.02	−0.07	0.13^*^	0.04	0.15^*^	0.10	0.06	−0.06	−0.09	0.16^**^		
15. Both	4.24 (0.53)	341	0.07	0.09	0.11	0.14^*^	−0.10	0.13^*^	0.07	0.24^***^	0.20^**^	0.11	−0.11	−0.11	0.37^***^	0.43^***^	
16. Exposure	2.76 (0.93)	345	−0.13^*^	0.02	0.01	−0.12^*^	−0.01	0.06	0.07	0.14^*^	0.04	−0.02	−0.01	0.02	0.09	0.43^***^	0.36^***^

Also shown in Table 2, greater levels of neuroticism were strongly related to higher levels of both state and trait anxiety and greater conscientiousness was positively related to job performance, or the participants’ perceptions of their job performance (self-efficacy). Additionally, higher levels of extraversion, openness to experience, and agreeableness were significantly related to lower levels of both state and trait anxiety. These constructs were all also significantly related to higher levels of counseling self-efficacy. That is, therapist participants who rated higher on extraversion, openness to experience, and agreeableness also had lower levels of anxiety and felt higher levels of self-efficacy in their jobs as clinicians.

## Discussion

This study provides data to foster implementation efforts for PTSD intervention by identifying the relative use of the various components of empirically supported treatments by community therapists. We also identified the characteristics that predict their use. Given the increasing importance of utilizing ESTs, a considerable amount of research has been published on which interventions are empirically supported for trauma. Reviews indicate roughly equivalent effectiveness of EMDR and CBT treatments for PTSD symptom reduction (Lewey et al., 2018). We found that only 16.31% of therapists in this study reported that it was “very true” that they followed a treatment manual closely, in line with previous research (Borntrager et al., 2009; Beidas and Kendall, 2010). In terms of the most common and least common treatment elements therapists are currently using in their practice, psychoeducation about trauma and traumatic experiences as well as emotion knowledge, affect identification, and emotion regulation or modulation skills were the two most commonly used treatment elements. This makes sense given that formalized protocols for EMDR are similar to CBT regarding providing psychoeducation and learning emotion regulation and coping skills (Schnyder et al., 2015). Deep breathing exercises or breathing training, increasing awareness of problem-solving skills and/or social skills, as well as addressing personal safety skills and assertive communication were also highly endorsed treatment components. Interestingly, explaining that the processing of trauma memories may continue after the therapy session, an explicit element of EMDR, was the third most commonly endorsed EST element. This shows that even therapists who do not use EMDR are informing their clients that processing of trauma memories may continue after their sessions together.

While only 135 participants reported being trained in EMDR therapy, 171 and 137 therapist participants, respectively, reported ‘always’ using two of its central treatment components: “reevaluation – check to make sure the client’s positive results have been maintained,” and “help establish a calm/safe space in their mind to ‘go’ when traumatic experiences are too much.” Potentially, EMDR developers may have “borrowed” these elements from CBT. However, we begin to ponder the question, despite having different underlying theoretical models, how different is EMDR from CBT in practice? In other words, are the components of the two modalities that different from each other?

Use of *in-vivo* exposure was the least commonly used treatment element in the current study consistent with previous research (Farrell et al., 2013; Harned et al., 2013). “I work with my clients to create a graded exposure hierarchy” and “I always work through the entire graded exposure hierarchy,” were also among the least endorsed treatment elements, with only 31 (8.66%) and 14 (3.90%) therapists endorsing “always” using them, respectively. This further lends credence to the idea that therapists consider EMDR to be like imaginal exposure. Perhaps only a small portion of those therapists trained in CBT are willing to use *in vivo* exposure, but therapists trained in both CBT and EMDR are willing to, or consider themselves, to use imaginal exposure. Greater “exposure” to the use of *in vivo* exposure may be needed in training programs (i.e., many therapists surveyed stated that they received their training in graduate school, and so it is possible that they did not have extensive training, including practice, with exposure sessions). Previous research has indicated that therapist training is related to the use of exposure, in particular that PhD psychologists use it most often (Whiteside et al., 2016), and this sample consisted of primarily individuals with a master’s degree in social work. Furthermore, females are also less likely to endorse the use of exposure (e.g., van Minnen et al., 2010) and our sample consisted of primarily female therapists.

The hypothesis that the CBT and EMDR items on the EST-Q would factor into at least two subscales – CBT and EMDR, was supported with the items loading onto CBT, EMDR, Both, and Exposure. While it appears that there are some EST elements that are unique to CBT and unique to EMDR, there are also several elements ant appear to fit in each modality. That is, there are elements of therapy that are used in both CBT and EMDR treatment modalities. The items on the “Both” CBT/EMDR subscale had the highest mean ratings. In terms of relative use of these four components EMDR therapists scored significantly higher than CBT or “Neither” therapists on the EMDR subscale, but not more than therapists cross-trained in “Both” CBT and EMDR. Therefore, those therapists that were trained in EMDR only, or those trained in EMDR and CBT, endorsed using more EMDR treatment elements more often than any other type of therapist. Moreover, “Both” therapists tended to have higher scores on the EMDR subscale than CBT therapists and “Neither” therapists.

While, therapists in the current study endorsed using EMDR primarily for treating clients with trauma exposure or PTSD symptoms (96.3%), therapists also endorsed using EMDR to treat clients with a range of presenting problems including anxiety disorders, depression, addiction, marital/relationship problems, health problems, and eating disorders. Moreover, participants who reported being trained in EMDR, also selected “other” and wrote in to the open ended section that they used EMDR to treat a range of other diagnoses, disorders, or problems including ADHD, dissociation, grief or loss, and dissociation. At this time, EMDR has not been established as efficacious for the treatment of depression (Carletto et al., 2017), bipolar disorder (Bedeschi, 2018), health problems (Cope et al., 2018; Dimitrov et al., 2019), or eating disorders (Balbo et al., 2017).

As expected, those with higher levels of both state and trait anxiety had lower levels of counseling self-efficacy (Larson et al., 1992) and clinicians who reported higher levels of extraversion, openness to experience, and agreeableness also had significantly lower levels of both state and trait anxiety, and higher levels of counseling self-efficacy. However, extraversion was not related to any of the four EST-Q subscales. Openness to experience was related (negatively) to the Exposure subscale, indicating that therapists who were more open were actually significantly less likely to endorse using exposure treatment components. Higher levels of conscientiousness were related to more usage of EMDR treatment components. Neuroticism was related to the EMDR subscale, and interestingly those who rated higher on neuroticism endorsed using significantly less EMDR treatment elements. Agreeableness was related to all but one (CBT) of the four EST-Q subscales. Higher levels of agreeableness were significantly related to more EMDR and “Both” treatment components, as well as less use of exposure treatment elements.

Therapists reporting higher trait anxiety reported they were less likely to endorse using EMDR treatment elements in their practice. This finding indicates that those therapists who are more anxious may feel less comfortable with, and therefore less likely to use, EMDR treatment components. Could it be that feelings of anxiety are causing therapists to choose more established or known therapies with their clients? Relatedly, overall counseling self-efficacy was significantly related to the EMDR and “Both” subscales. That is, therapists with greater overall counseling self-efficacy were also significantly more likely to endorse using EMDR and Both treatment components. Therefore, the hypothesis that therapists with higher anxiety and lower self-efficacy may be less likely to use EST components was partially supported.

Findings point to the possibility that EMDR trained therapists are using the empirically supported EMDR components at a greater rate than CBT trained therapists are to CBT components, when it comes to the treatment of trauma. Furthermore, with very few therapists endorsing the use of exposure, it is unlikely that therapists are providing exposure-based CBT in a manner consistent with clinical practice guidelines. Findings are also consistent with past research indicating that therapists are concerned about client distress during exposure and also report high levels of personal distress during exposure-based strategies (e.g., Zoellner et al., 2011). EMDR does not require *in vivo* exposure or detailed verbalization of traumatic events, and the current results provide support for the notion that EMDR is more palpable for therapists. Thus, it is perhaps time to consider supplemental training, alongside training in exposure-based techniques, that addresses these concerns about both client and therapist distress during exposure. For example, therapists’ own use of emotion regulation skills and other coping strategies may facilitate the adoption and more frequent use of exposure techniques. Implementation research efforts are needed to identify clinician friendly predictors of favorable outcomes with various packages of components or at smaller dosages of intervention as well as assessable predictors of those who might benefit from abbreviated protocols or certain packages of components (Weems, 2019). An additional avenue is identifying the downstream effects of intervention targeting on one or more core symptoms on overall improvement (Weems, 2020).

Finally, previous work has primarily focused on doctoral-level psychologists, despite this group being only 16% of mental health professionals (Hamp et al., 2014), and thus little is known about EST use among the other professions that provide the majority of community PTSD treatment services. Thus, the current study provides much needed information about EST use among social workers and clinicians with masters degrees, and indicates that EMDR is becoming increasingly popular among these mental health professionals.

### Limitations and future directions

Findings should be understood in terms of the study limitations. First, the sample consists of only those who had their email or mailing (in the case of LMFTs) address available from a state or national database. Another limitation of this study was the use of self-report measures. Since therapists tend to overestimate their clinical skills (Parker and Waller, 2015; Waller and Turner, 2016) it is possible that self-report measures of both counseling self-efficacy and use of empirically supported treatment elements may be overestimated by study participants. From more diversity in the therapist sample, to more diversity in a client sample, diverse populations should be sought in any future studies. It appears that the evidence for what white female therapists are doing is growing, but we know less about therapists in the US who are not white and do not identify as female. In addition, while the role of “therapeutic experience” was examined by controlling for this variable (see Supplementary Tables C1–C4) the role of experience may exhibit differential effects on technique use over time that may only be evident in a longitudinal design. Following therapist use of empirically supported treatments over time could provide valuable insights beyond this study. Finally, the labeling of factors and interpretation of results is limited to what we measured. As noted we aimed to examine both EMDR and CBT components; however, the results may not be a true indicator of what CBT and EMDR actually looks like in usual care. That is our results should not be interpreted as meaning these are the only four aspects of these therapies that are used together. Person centered analyses looking at clusters of therapist use of various components could help address better “types of therapists.” Similarly, the “Both” factor should be evaluated with a lens toward how these items practically (i.e., in the practice setting) differ from items on either of the other three factors. As with any self-report interpretation of the items may influence results and differ from actual practice.

Although EST’s exist and are supported by major professional organizations, the majority of clients in treatment do not receive them (Beidas and Kendall, 2010). More and better clinician training on EST’s is viewed by many as a key to improving client care (Garland et al., 2010). Lack of time and resources for training, as well as lack of supervision and consultation, are frequently cited as reasons that implementing EST’s in community settings does not work (Garland et al., 2010). Barriers to implementing EST’s include time constraints, excessive paperwork, and lack of reimbursement for activities related to implementing EST’s such as training and supervision (Riemer et al., 2005). Addis et al. (1999) cite several reasons why clinicians are hesitant to use empirically-supported, manualized treatments including effects on the therapeutic relationship, unmet client needs, competence and job satisfaction, treatment credibility, hindering clinician innovation, and the feasibility of manualized treatments.

There is evidence that therapists who have a more favorable opinion of ESTs are more likely to use them (Kolko et al., 2009). Borntrager et al. (2009) conducted a study to investigate whether clinicians had a problem with the evidence or the manuals for EST’s. They found that after completing training for an EST, therapists’ views of EST’s were more positive. However, they also found that there was a significant difference in how the therapists felt about the EST’s based on how they were asked. Therapists who took a survey where the term “manual” was used had significantly less favorable view of EST’s compared with therapists who took a survey that deemphasized the word “manual” (Borntrager et al., 2009). The terminology used by researchers may impact community therapists’ willingness to use EST manuals or manual components.

In the case of EMDR, training is intensive and can be costly. Programs such as the EMDR Humanitarian Assistance Program (EMDR HAP) have been created to try to reduce the cost and get EMDR into the hands of more clinicians. However, this course requires two separate three-day weekends of training, with time needed for practice in between weekends. After training, clinicians must then practice EMDR and usually pay for supervision from a certified EMDR consultant in order to obtain their certificate to practice EMDR independently. However, the condensed nature of the training in only six days and over weekends, allows some clinicians the time to complete the training. Additionally, there is only one treatment manual for EMDR – it is uniform, and anyone trained in EMDR will be trained in the same way, no matter where they received their training. Finally, many non-profits and other organizations bring trainings such as EMDR HAP to their employees and pay for the course, others offer reimbursement for training, and many offer free supervision from EMDR consultants within their own organization or for free or at a low cost with other trainers (EMDR Humanitarian Assistance Programs, 2017).

Qualitative studies may be one way to bridge the gap between research and practice, by gathering more information directly from practitioners in the field (Kazdin and Weisz, 1998). In a qualitative study using focus groups to get provider feedback on implementing a specific EST for children and adolescents, Reding et al. (2016) found there were several themes that emerged. Many practitioners appreciated that the program incorporated flexibility to be able to adapt the intervention to their clients’ needs (Reding et al., 2016). However, clinicians also wanted more time to build a therapeutic relationship with their clients instead of needing to begin with treatment modules right away (Reding et al., 2016), a concern that echoes other practitioner apprehensions described above. These focus groups also echoed concerns about system-level challenges of implementing the treatment in a community-based setting, such as time constraints and training taking up practitioner time (Reding et al., 2016).

Exposure sessions for PTSD have the most empirical evidence, but are also the most underused treatment (Farrell et al., 2013; Harned et al., 2013). Reasons for this, as cited by therapists, range from exposure carrying a high risk of harm to clients, to clients being unable to tolerate exposure, therapist characteristics such as anxiety and self-efficacy, but mostly, lack of use of EST’s, and exposure sessions specifically, is related to therapist training (Foy et al., 1996). Research has shown the key barriers to using exposure therapy in practice are in fact therapist factors, and not client factors. Armed with this knowledge, the field may be able to make changes to therapist education and training in order to increase EST use and quality in community practice.

## Conclusion

In conclusion, this study identified relative use of EST components for CBT and EMDR. EMDR therapists reported higher mean scores on their EMDR subscale (i.e., greater relative use of the core EMDR techniques) than CBT therapists. However, “off label” use of components was also identified with application of EMDR techniques to other diagnoses not yet supported in the literature. The data also found an underutilization of *in vivo* exposure techniques. Results identify therapist characteristics that predict the use of various techniques suggesting avenues for implementation efforts to address therapist reluctance to use certain components or techniques. The findings suggest implementation efforts develop techniques to improve the use of underused techniques and address barriers to their utilization in training programs. Empirical knowledge of the outcomes associated with various packages of components or at smaller dosages of intervention as well as clinician assessable predictors of those who might benefit from abbreviated protocols or certain packages of components is also a potential line of future inquiry in this regard.

## Data availability statement

The raw data supporting the conclusions of this article will be made available by the authors, without undue reservation.

## Ethics statement

Iowa State University Office for Responsible Research’s Institutional Review Board reviewed and approved this study (IRB #17-204). The studies were conducted in accordance with the local legislation and institutional requirements. The participants provided their written informed consent to participate in this study.

## Author contributions

EN: conceptualization, data collection, curation, analysis, writing, and editing. AZ and CW: conceptualization, writing, and editing. All authors contributed to the article and approved the submitted version.
