# Supplementary material for: Therapist use of cognitive behavior therapy and eye movement desensitization and reprocessing components for the treatment of posttraumatic stress disorder in practice settings
Source: Front Psychol. 2023 Oct 19;14:1158344. doi: 10.3389/fpsyg.2023.1158344 (PMC10621788; doi:10.3389/fpsyg.2023.1158344)
Supplement: Supplementary file 1 [file Data_Sheet_1.PDF]

Supplemental Information for Community therapist use of CBT and EMDR components for the treatment of PTSD

.

Supplement A: Details of efforts to recruit therapists and detailed demographic information

Emails were sent to the various State/Provincial/City Chapters of the American Psychological Association and National Association of Social Workers Contacted to participate in the study and 462 responded and agreed to participate. Of those 462 potential participants who responded agreed to the informed consent, 434 (93.94%) answered the study inclusion questions that they were a licensed therapist who spends at least 50% of their professional time seeing clients for individual therapy (both inclusion criteria questions). Sixteen potential participants (3.46%) were excluded from the study because they did not meet inclusion criteria and twelve potential participants (2.60%) did not answer the question regarding inclusion criteria, and therefore were not able to continue onto the survey.

Table A1. *State/Provincial/City Chapters of the American Psychological Association and National Association of Social Workers Contacted to Participate in the Study Survey, Their Response, and Distribution Method(s), If Applicable*

State	APA	NASW	State	APA	NASW
Alabama	X	X	Montana	N	X
Alaska	X	N	Nebraska	N	X
Arizona	X	N	Nevada	N	X
Arkansas	X	X	New Hampshire	Y	Y – newsletter
California	N	X	New Jersey	N – fee involved	X
Colorado	Y – email	N	New Mexico	X	X
Connecticut	X	Y – email	New York City	N/A	X
Delaware	Y	X	New York State	X	X
DC	Y – email	X	North Carolina	X	X
Florida	N	N	North Dakota	Y – email	X
Georgia	N	X	Ohio	Y – email	Y – email newsletter
Guam	N/A	X	Oklahoma	N	X
Hawaii	X	X	Oregon	Y – listserv	Y – social media/website

Idaho	N	Y – social media	Pennsylvania	N	X
Illinois	N	X	Rhode Island	X	N
Indiana	N	N	South Carolina	X	Y – Facebook
Iowa	Y – email/ newsletter	Y – email	South Dakota	X	X
Kansas	Y – email	X	Tennessee	Y – website	X
Kentucky	X	Y – Facebook	Texas	Y – email	X
Louisiana	X	Y – website and Facebook	Utah	X	X
Maine	Y – email	X	Vermont	Y – listserv	X
Maryland	N	X	Virgin Islands	n/a	X
Massachusetts	X	X	Virginia	N	X
Michigan	X	N – must purchase listserv	Washington state	Y – email	X
Minnesota	Y	N	West Virginia	X	X
Mississippi	N	N – fee involved	Wisconsin	X	Y – email
Missouri	X	N – fee involved	Wyoming	Y – email	Y – social media

Table A1. *Continued*

Province	APA	NASW
Alberta	Yes – website	n/a
Manitoba	No – fee involved	n/a
Nova Scotia	Yes – email	n/a
Ontario	X	n/a
Québec	X	n/a
British Columbia	X	n/a

Note. X denotes that the state chapter gave no response to the two email contacts from the study author. N/A denotes that such a chapter does not exist in that organization. APA = American Psychological Association. NASW = National Association of Social Workers

Table A2. *Regional Chapters of the EMDR International Association Contacted to Participate in the Study Survey, Their Response, and Distribution Methods, If Applicable.*

**EMDRIA Regional Chapter**	**Response**
North Alabama	X
South Alabama	No listserv exists; sent to members and colleagues
Alaska	X
Central and Northern Arizona	X
Southern Arizona	Not able to contact – email returned
Arkansas	X
California Central Valley	Yes – email
Chico California	Yes – email
Greater Sacramento	Yes – Facebook and email
Los Angeles County	No
Northern California	X
San Diego County	X
San Francisco/South Bay	No
Santa Cruz County	X
Santa Maria California	X
Southern California	X
Superior Northern California	X
Boulder	Yes – Facebook
Denver	X
Northern Colorado	No to listserv; passed onto people they knew
Pikes Peak	Facebook group; ELN posted twice
Western Colorado	X
Fairfield County/Southwestern CT	X
New Haven	No – steering committee said too many survey questions were not about EMDR
Northern Connecticut	X
Northwest CT	X
Greater Orlando	Yes – Facebook
Northwest Florida	No email contact information
Southeast Florida	Yes – email
Tampa Bay	X
Georgia	X

Table A2. *Continued.*

EMDRIA Regional Chapter	Response
Eastern Oregon and Idaho	X
Chicago	Yes – email
Greater Louisville	Yes – Facebook
Indianapolis	X
NE/Northern Indiana	Yes – Facebook
Central Iowa	No – group is not currently active
Northwest Iowa	Yes – email
Greater Kansas City	X
Southcentral Kansas	X
South Louisiana	Yes – Facebook
Southern Maine	Yes – email
Greater Baltimore-Washington	X
Tri-State (MD, DE, PA)	No formal organization; will send to 35 people they know
Berkshire County MA	X
Boston/West Suburban	No
Greater Boston	Yes – email
Western Mass	X
Michigan and Northwest Ohio	No
Michigan	X
Minnesota	No – organization is too new
Mississippi	X
Tennessee and Northern Mississippi	X
Central Missouri	No – organization is too new
Lake of the Ozarks	X
St. Louis	Yes – Yahoo group and email
Nebraska-Great Plains	Yes – Facebook
Southeast Nebraska	Yes – email
Northern Nevada	X
Southern Nevada	X
New Jersey	X
New Mexico	X
Central New York	X
Long Island	No

Table A2. *Continued*

EMDRIA Regional Chapter	Response
New York City	X
Westchester County NY	X
Western New York	Yes – email
Asheville NC	X
Greater Charlotte Area	X
North Carolina	No
Western North Carolina	X
North Dakota	X
Greater Cincinnati	Yes – email
Greater Cleveland	X
Mid-Ohio	No email contact information
Northeast Ohio	X
Northwest Ohio	X
Oklahoma	X
Central Oregon	Yes – email
Portland	Yes – email
Southern Oregon	X
Central Pennsylvania	No
Montgomery-Bucks County	Did not contact
Philadelphia	X
Rhode Island	X
South Dakota	Yes – website
East Tennessee	No
Middle Tennessee	X
Big Country	X
Central Texas	X
DFW West/Metroplex East	Yes – email and Facebook groups
Far West Texas	X
Greater Houston Area	No listserv
Heart of Texas	No
South Texas	Yes – email
Southern Utah	Yes – email
Tri-County Area Utah	X
Utah	No email contact information
Vermont	No

Table A2. *Continued*

EMDRIA Regional Chapter	Response
Central Virginia	X
Northern Virginia	No
Rappahannock Area	X
Central Washington	X
Eastern Washington	X
NW Washington	No
SW Washington	X
Wisconsin	No listserv
Wyoming	X

Note: X denotes that the state chapter gave no response to the two email contacts from the study author. EMDRIA = Eye Movement Desensitization and Reprocessing International

Table A3. *Additional Participant Demographics*

	Mean (SD)	
Age (*n* = 338)	44.59 (12.99)	
# of Individual Therapy Clients per Week	21.03 (8.10)	
Years Since Graduate Degree (*n* = 340)	13.26 (10.77)	
		N (%)
Gender (*n* = 339)	Female	285 (84.07%)
	Male	51 (15.04%)
	Non-binary/third gender	2 (0.59%)
	Prefer not to answer	1 (0.29%)
Race (*n* = 338)	White	294 (86.98%)
	Hispanic or Latino	16 (4.73%)
	Prefer to Self-Describe	10 (2.96%)
	Prefer Not to Answer	9 (2.66%)
	Black or African American	6 (1.78%)
	Asian	3 (0.89%)
Highest Degree Held (*n* = 339)	Master's Degree	226 (66.67%)
	Doctoral Degree	109 (32.15%)
	Other	4 (1.18%)
Type of License (*n* = 338)	Licensed Clinical Social Worker	125 (36.98%)
	Licensed Psychologist	84 (24.85%)
	Licensed Professional Counselor	54 (15.98%)
	Other	43 (12.72%)
Type of License – "Other" (*n* = 43)	Licensed Marriage and Family Therapist	32 (9.47%)
	Licensed Mental Health Counselor	19 (5.62%)

Table A3. *Continued.*

Years Practicing as a Therapist (*n* = 341)	5 years or less	87 (25.51%)
	6-10 years	103 (30.21%)
	11-15 years	35 (10.26%)
	16-20 years	42 (12.32%)
	21-25 years	26 (7.62%)
	26-30 years	15 (4.40%)
	31-35 years	14 (4.11%)
	35 years or more	19 (5.57%)
Primary Employment Setting (*n* = 340)	College/University	6 (1.76%)
	Community Clinic/Agency	82 (24.12%)
	Managed Care Organization	7 (2.06%)
	Primary/secondary school	3 (0.88%)
	Private Practice	195 (57.35%)
	Hospital	25 (7.35%)
	Other	22 (6.47%)
Type of Clients Primarily Seen (*n* = 338)	Children (ages 3-11)	32 (9.47%)
	Adolescents (ages 12-18)	49 (14.50%)
	Emerging Adults (ages 19-27)	37 (10.95%)
	Adults (ages 28-65)	216 (63.91%)
	Older Adults (ages 65+)	4 (1.18%)
Theoretical Orientation (*n* = 257)	Other	70 (27.24%)
	Eclectic	65 (25.29%)
	Family Systems	55 (21.40%)
	CBT	25 (9.73%)
	Interpersonal	22 (8.56%)
	Psychodynamic/Analytic	8 (3.11%)
	Existential/Humanistic	5 (1.95%)
	Experiential	4 (1.56%)
	Social Learning	2 (0.78%)
	Behavioral	1 (0.39%)

Table A4. *Participant Report of State(s) of Licensure.*

Chapter	*N*	%	Chapter	*n*	%
**Alabama**	1	0.28%	**Oregon**	10	2.75%
**Alaska**	0	-	**Pennsylvania**	15	4.13%
**Arizona**	4	1.53%	**Puerto Rico**	0	-
**Arkansas**	1	0.28%	**Rhode Island**	0	-
**California**	17	4.68%	**South Carolina**	4	1.53%
**Colorado**	16	4.41%	**South Dakota**	0	-
**Connecticut**	8	2.20%	**Tennessee**	3	0.83%
**Delaware**	1	0.28%	**Texas**	22	6.06%
**DC**	6	1.65%	**Utah**	9	2.48%
**Florida**	6	1.65%	**US Virgin Islands**	0	-
**Georgia**	3	0.83%	**Vermont**	1	0.28%
**Hawaii**	2	0.55%	**Virginia**	13	3.58%
**Idaho**	0	-	**Washington**	3	0.83%
**Illinois**	13	3.58%	**West Virginia**	0	-
**Indiana**	4	1.53%	**Wisconsin**	16	4.41%
**Iowa**	67	18.46%	**Wyoming**	1	0.28%
**Kansas**	4	1.53%	**TOTAL**	363	
**Kentucky**	0	-			
**Louisiana**	11	3.03%			
**Maine**	1	0.28%	**OUTSIDE US TOTAL**	10	
**Maryland**	6	1.65%	**Canada**	2	20%
**Massachusetts**	10	2.75%	**Alberta**	0	-
**Michigan**	6	1.65%	**Manitoba**	0	-
**Minnesota**	8	2.20%	**Nova Scotia**	2	20%
**Mississippi**	0	-	**Ontario**	1	10%
**Missouri**	5	1.38%	**Québec**	0	-
**Montana**	3	0.83%	**British Columbia**	0	-
**Nebraska**	6	1.65%	**OTHER**		
**Nevada**	0	-	**Italy**	1	10%

**New Hampshire**	0	-	**Netherlands**	1	10%
**New Jersey**	3	0.83%	**missing**	3	30%
**New Mexico**	1	0.28%			
**New York**	25	6.89%			
**North Carolina**	5	1.38%			
**North Dakota**	3	0.83%			
**Ohio**	17	4.68%			
**Oklahoma**	3	0.83%			

Note: Participants may be licensed in more than one state (/jurisdiction).

Supplement B Additional data, data tables, and analytic information for main analyses

Table B *The 32 EST Components of the Empirically Supported Treatment Questionnaire by Protocol*

	**CBT**	**EMDR**
1.	Use of a standard measure prior to session to assess client's level of symptoms for the day's session	Identify processing targets from positive and negative events in client's life (i.e. first or worst traumatic event)
2.	Agenda setting – articulate & implement a specific agenda for session, identify other issues	Have the client imagine a container to hold memories/thoughts when not working through them
3.	Review with client previous homework – praise efforts and troubleshoot obstacles	Provide client an explanation of Eye Movement Desensitization and Reprocessing
4.	Utilize homework and other educational materials – informational handouts, worksheets, etc. with client	Help client establish a calm/safe place in their mind to "go" when traumatic memories are too much
5.	Deep breathing exercises or breathing training	Establish a stop signal for when traumatic memories are too much to continue processing/end of session
6.	Use of guided imagery/imaginal exposure	Have client do body scan (i.e. "Where do you feel the trauma in your body?")
7.	Work on emotion knowledge/affect identification and emotion regulation/modulation skills	Elicit image of the traumatic event, negative belief currently held, desired positive belief, current emotion(s), and physical sensation (body location)
8.	Use of cognitive restructuring with client (thought-feeling model, connect negative feelings to thoughts, challenge thoughts, generate alternative thought, practice alternative thoughts)	Use of Validity of Positive Cognition ("VOC") "How true do those words ____feel to you now?"
9.	Assign thought record or daily diary to client (client to record thoughts, feelings/emotions, behaviors/actions)	Use of Subjective Units of Disturbance Scale ("SUDS") "How disturbing does it feel to you now?"
10.	Help client develop a trauma narrative	Bilateral stimulation with negative cognition and traumatic event (e.g. eye movements, tactile or visual stimulation, etc.)
11.	I work with my clients to create a graded exposure hierarchy	Bilateral stimulation with positive cognition (e.g. eye movements, tactile or visual stimulation, etc.)
12.	I always work through the entire graded exposure hierarchy	Use "Cognitive Interweave" to open blocked processing by elicitation of more adaptive information

Table B. *Continued*

	**CBT**	**EMDR**
13.	Use of in-vivo exposure	Explain that processing of trauma memories may continue after the session
14.	Address personal safety skills and assertive communication	Reevaluation – Check to make sure the client's positive results have been maintained
15.	Increase awareness of problem-solving skills and/or social skills	Psychoeducation – provide the client information about traumatic experiences, trauma reactions, symptoms, and trauma reminders *
16.	Use of homework assigning (e.g. develop homework assignment, collaborate with client, make specific plan, troubleshoot obstacles)	Provide progressive muscle relaxation (or provide other progressive relaxation skills) *

* Denotes items that were common to both CBT and EMDR

Table B1. *Frequency Table of EST-Q Items for Those Therapists Trained in CBT – Essential EMDR and CBT Elements Presented to CBT Trained Therapists*

			Frequency				
	M (*SD*)	*n*	Never	Rarely	Sometimes	Often	Always
Psychoeducation – provide the client information about traumatic experiences, trauma reactions, symptoms, and trauma reminders	4.69 (0.59)	272	0	2	12	54	204
Work on emotion knowledge/affect identification and emotion regulation/modulation skills	4.46 (0.69)	272	0	4	18	98	152
Deep breathing exercises or breathing training	4.42 (0.78)	272	2	4	26	86	154
Explain that processing of trauma memories may continue after the session	4.41 (0.85)	272	4	8	18	85	157
Address personal safety skills and assertive communication	4.27 (0.80)	271	1	7	32	110	121
Increase awareness of problem-solving skills and/or social skills	4.25 (0.83)	270	1	8	37	100	124
Reevaluation – Check to make sure the client's positive results have been maintained	4.23 (0.89)	272	5	6	36	99	126
Use of cognitive restructuring with client (thought-feeling model, connect negative feelings to thoughts, challenge thoughts, generate alternative thought, practice alternative thoughts)	4.10 (0.92)	272	3	10	54	95	110
Review with client previous homework – praise efforts and troubleshoot obstacles	4.00 (0.96)	272	7	14	42	118	91
Utilize homework and other educational materials – informational handouts, worksheets, etc. with client	3.96 (0.93)	272	4	14	57	112	85
Use of guided imagery/imaginal exposure	3.94 (0.98)	271	8	14	48	118	83

Table B1. *Continued.*

	M (*SD*)	*n*	*Frequency* Never	Rarely	Sometimes	Often	Always
Help client establish a calm/safe place in their mind to "go" when traumatic memories are too much	3.89 (1.17)	272	17	19	43	91	102
Use of homework assigning (e.g. develop homework assignment, collaborate with client, make specific plan, troubleshoot obstacles)	3.81 (0.98)	271	6	20	64	110	71
Provide progressive muscle relaxation (or provide other progressive relaxation skills)	3.77 (0.93)	272	6	18	67	123	58
Have client do body scan (i.e. "Where do you feel the trauma in your body?")	3.72 (1.11)	271	13	25	63	95	75
Agenda setting – articulate & implement a specific agenda for session, identify other issues	3.69 (1.06)	272	12	22	68	105	65
Identify processing targets from positive and negative events in client's life (i.e. first or worst traumatic event)	3.69 (1.16)	271	21	17	61	99	73
Elicit image of the traumatic event, negative belief currently held, desired positive belief, current emotion(s), and physical sensation (body location)	3.62 (1.20)	271	21	26	59	93	72
Help client develop a trauma narrative	3.57 (1.10)	270	18	20	77	100	55
Establish a stop signal for when traumatic memories are too much to continue processing/end of session	3.56 (1.31)	272	29	29	57	74	83
Use of Subjective Units of Disturbance Scale ("SUDS") "How disturbing does it feel to you now?"	3.45 (1.39)	272	40	29	51	72	80
Assign thought record or daily diary to client (Client to record thoughts, feelings/emotions, behaviors/actions)	3.12 (0.98)	271	13	57	106	75	20
Use of a standard measure prior to session to assess client's level of symptoms for the day's session	2.97 (1.29)	271	45	58	65	67	36

Table B1. *Continued.*

	M (*SD*)	*n*	*Frequency*				
			Never	Rarely	Sometimes	Often	Always
Have the client imagine a container to hold memories/thoughts when not working through them	2.96 (1.42)	272	64	41	54	68	45
I work with my clients to create a graded exposure hierarchy.	2.86 (1.25)	272	53	49	77	68	25
Use of Validity of Positive Cognition ("VOC") "How true do those words ____feel to you now?"	2.86 (1.45)	272	71	48	49	57	47
Provide client an explanation of Eye Movement Desensitization and Reprocessing	2.68 (1.70)	272	119	24	22	40	67
I always work through the entire graded exposure hierarchy.	2.51 (1.17)	272	68	67	80	44	13
Use "Cognitive Interweave" to open blocked processing by elicitation of more adaptive information	2.51 (1.49)	272	114	24	48	53	33
Use of in-vivo exposure	2.40 (1.19)	272	80	72	62	47	11
Bilateral stimulation with negative cognition and traumatic event (e.g. eye movements, tactile or visual stimulation, etc.)	2.37 (1.61)	272	147	11	21	53	40
Bilateral stimulation with positive cognition (e.g. eye movements, tactile or visual stimulation, etc.)	2.32 (1.58)	354	149	12	24	50	37

Table B2. *Frequency Table of EST-Q Items for Those Therapists Trained in EMDR – Essential EMDR and CBT Elements Presented to EMDR Trained Therapists*

	M (*SD*)	*n*	*Frequency*				
			Never	Rarely	Sometimes	Often	Always
Psychoeducation – provide the client information about traumatic experiences, trauma reactions, symptoms, and trauma reminders	4.78 (0.48)	135	0	0	4	22	109
Explain that processing of trauma memories may continue after the session	4.67 (0.60)	135	0	2	3	32	98
Help client establish a calm/safe place in their mind to "go" when traumatic memories are too much	4.49 (0.65)	135	1	5	55	74	0
Reevaluation – Check to make sure the client's positive results have been maintained	4.47 (0.77)	135	1	2	11	39	82
Work on emotion knowledge/affect identification and emotion regulation/modulation skills	4.46 (0.78)	135	1	3	9	42	80
Provide client an explanation of Eye Movement Desensitization and Reprocessing	4.46 (0.84)	135	3	1	9	40	82
Deep breathing exercises or breathing training	4.42 (0.74)	135	1	1	11	49	73
Have client do body scan (i.e. "Where do you feel the trauma in your body?")	4.42 (0.76)	135	1	2	10	48	74
Establish a stop signal for when traumatic memories are too much to continue processing/end of session	4.32 (0.94)	135	3	3	17	37	75
Elicit image of the traumatic event, negative belief currently held, desired positive belief, current emotion(s), and physical sensation (body location)	4.27 (0.82)	135	2	1	17	54	61
Use of Subjective Units of Disturbance Scale ("SUDS") "How disturbing does it feel to you now?"	4.26 (0.92)	135	2	5	17	43	68
Address personal safety skills and assertive communication	4.19 (0.84)	135	1	4	19	55	56
Increase awareness of problem-solving skills and/or social skills	4.19 (0.85)	135	0	4	26	45	60

Table B2. *Continued.*

	M (*SD*)	*n*	*Frequency*				
			Never	Rarely	Sometimes	Often	Always
Bilateral stimulation with negative cognition and traumatic event (e.g. eye movements, tactile or visual stimulation, etc.)	4.14 (0.86)	135	3	2	17	64	49
Identify processing targets from positive and negative events in client's life (i.e. first or worst traumatic event)	4.10 (0.93)	134	3	5	18	58	50
Bilateral stimulation with positive cognition (e.g. eye movements, tactile or visual stimulation, etc.)	4.06 (0.93)	135	4	4	18	63	46
Use of Validity of Positive Cognition ("VOC") "How true do those words ____feel to you now?"	4.05 (1.08)	135	5	9	18	45	58
Use of guided imagery/imaginal exposure	4.04 (0.93)	134	2	6	24	54	48
Have the client imagine a container to hold memories/thoughts when not working through them	4.01 (1.03)	135	6	4	22	54	49
Review with client previous homework – praise efforts and troubleshoot obstacles	3.93 (1.02)	135	5	7	23	57	43
Agenda setting – articulate & implement a specific agenda for session, identify other issues	3.84 (1.00)	135	4	9	29	56	37
Utilize homework and other educational materials – informational handouts, worksheets, etc. with client	3.74 (1.02)	135	3	10	43	42	37
Provide progressive muscle relaxation (or provide other progressive relaxation skills)	3.74 (0.86)	135	0	11	39	59	26
Use of cognitive restructuring with client (thought-feeling model, connect negative feelings to thoughts, challenge thoughts, generate alternative thought, practice alternative thoughts)	3.73 (0.93)	135	3	7	42	54	29
Use "Cognitive Interweave" to open blocked processing by elicitation of more adaptive information	3.71 (1.15)	135	10	8	29	52	36

Table B2. *Continued.*

	M (*SD*)	*n*	*Frequency*				
			Never	Rarely	Sometimes	Often	Always
Use of homework assigning (e.g. develop homework assignment, collaborate with client, make specific plan, troubleshoot obstacles)	3.47 (1.00)	135	5	15	47	48	20
Help client develop a trauma narrative	3.42 (1.08)	135	10	10	50	43	22
Use of a standard measure prior to session to assess client's level of symptoms for the day's session	2.87 (1.30)	134	25	33	25	37	14
Assign thought record or daily diary to client (Client to record thoughts, feelings/emotions, behaviors/actions)	2.78 (0.95)	135	11	42	52	26	4
I work with my clients to create a graded exposure hierarchy.	2.67 (1.24)	135	33	24	41	28	9
I always work through the entire graded exposure hierarchy.	2.39 (1.13)	135	37	36	40	17	5
Use of in-vivo exposure	2.16 (1.07)	135	46	40	35	10	4

Additional Details of the factor analysis

Missing data was handled by pairwise deletion, as acceptable given the limited missingness in the data (Tabachnick & Fidell, 2001) leaving 329 cases for analysis. This is an appropriate sample size as there were 32 items on the EST-Q, and at least 5-10 participants per item is recommended (Russell, 2002). The goal of the analysis was to determine the most theoretically consistent structure while minimizing cross loadings and parsimony in the number of factors. Kaiser's criterion (i.e., eigenvalues greater than one) was initially used as the primary criterion for selecting the number of factors, then confirmed through visual inspection of a scree plot (Preacher & MacCallum, 2003). Examination of the amount of variance explained by the factors was also employed and the consistency of item loadings with theory and predictions (i.e., that the items would factor into unique EMDR and CBT components) were also considered. Loadings of .30 or greater were considered salient. Given the goals of analysis and theoretical considerations, principal factors extraction with direct oblimin rotation was used (Tabachnick & Fidell, 2001). The factorability of the items appeared appropriate given the pattern of correlations among the items, as many inter-item correlations exceeded .30. Coefficient alpha was used to assess internal consistency (Tabachnick & Fidell, 2001).

Seven factors with eigenvalues over one were extracted and accounted for a total of 65.84% of the total variance. Examination of the Scree plot, however, did not support a seven-factor solution. The Scree plot appeared to have a break between four and five factors. The analysis was then repeated, forcing a four-factor solution consistent with theory and hypotheses. As noted this four-factor model appeared to be the best fit conceptually and accounted for 55.26% of the total variance. The four subscales that emerged all had

good internal consistency (> .75) and were named CBT (α = .80), EMDR (α = .93), Both (α = .77; a factor containing items that are representative of both CBT and EMDR modalities), and exposure (α = .81; a factor for items relating to exposure techniques). These labels should not be interpreted as meaning these are the only four aspects of these therapies that are used together or that these items are fully core to these therapies – simply that these are where these items loaded in the analysis and it makes sense given where the various items came from to label these as such. Factor loadings are available in Supplemental Table B3 below.

Russell, D. W. (2002). In search of underlying dimensions: The use (and abuse) of factor analysis in Personality and Social Psychology Bulletin. Personality and Social Psychology Bulletin, 28(12), 1629–1646.

Tabachnick, G., & Fidell, L. (2001). Using multivariate statistics [4th ed]. Boston: Allyn & Bacon.

Table B3. *Factor Loadings of the EST-Q*

	Factor			
EST-Q Item	*EMDR*	*CBT*	*Both*	*Exposure*
Bilateral stimulation with negative cognition and traumatic event (e.g. eye movements, tactile or visual stimulation, etc.)	**.917**	—	—	—
Bilateral stimulation with positive cognition (e.g. eye movements, tactile or visual stimulation, etc.)	**.908**	—	—	—
Use of Validity of Positive Cognition ("VOC"): "How true do those words ____feel to you now?"	**.823**	—	—	—
Provide client an explanation of Eye Movement Desensitization and Reprocessing	**.810**	—	—	—
Use "Cognitive Interweave" to open blocked processing by elicitation of more adaptive information	**.758**	—	—	—
Have the client imagine a container to hold memories/thoughts when not working through them	**.717**	—	.307	—
Have client do body scan (i.e. "Where do you feel the trauma in your body?")	**.691**	—	.372	
Elicit image of the traumatic event, negative belief currently held, desired positive belief, current emotion(s), and physical sensation (body location)	**.675**	—	—	.327
Establish a stop signal for when traumatic memories are too much to continue processing/end of session	**.667**	—	.449	
Use of Subjective Units of Disturbance Scale ("SUDS") "How disturbing does it feel to you now?"	**.653**	.332	—	.446
Help client establish a calm/safe place in their mind to "go" when traumatic memories are too much	**.591**	—	.486	
Identify processing targets from positive and negative events in client's life (i.e. first or worst traumatic event)	**.541**	.433	—	.426
Reevaluation – Check to make sure the client's positive results have been maintained	**.423**		.415	—
Use of homework assigning (e.g. develop homework assignment, collaborate with client, make specific plan, troubleshoot obstacles)	—	**.812**	—	—

Table B3. *Continued.*

EST-Q Item	*Factor* *EMDR*	*CBT*	*Both*	*Exposure*
Utilize homework and other educational materials – informational handouts, worksheets, etc. with client	—	**.765**	—	—
Review with client previous homework – praise efforts and troubleshoot obstacles	—	**.748**	—	.360
Assign thought record or daily diary to client (client to record thoughts, feelings/emotions, behaviors/actions)	—	**.579**	—	.331
Agenda setting – articulate & implement a specific agenda for session, identify other issues	.380	**.520**	—	.358
Use of cognitive restructuring with client (thought-feeling model, connect negative feelings to thoughts, challenge thoughts, generate alternative thought, practice alternative thoughts)	—	**.480**	.372	—
Use of a standard measure prior to session to assess client's level of symptoms for the day's session	—	**.398**	—	.327
Address personal safety skills and assertive communication	—	—	**.591**	—
Increase awareness of problem-solving skills and/or social skills	—	—	**.589**	—
Deep breathing exercises or breathing training	—	—	**.571**	—
Explain that processing of trauma memories may continue after the session	.410	—	**.547**	—
Provide progressive muscle relaxation (or provide other progressive relaxation skills)	—	.382	**.501**	.361
Use of guided imagery/imaginal exposure	.345	.401	**.486**	.392
Work on emotion knowledge/affect identification and emotion regulation/modulation skills	—	—	**.439**	—
Help client develop a trauma narrative	—	. —	**.362**	.359
Psychoeducation – provide the client information about traumatic experiences, trauma reactions, symptoms, and trauma reminders	—	—	**.344**	—
I work with my clients to create a graded exposure hierarchy.	—	.364	—	**.882**
I always work through the entire graded exposure hierarchy.	—	.419	—	**.848**
Use of in-vivo exposure	—	.346	—	**.741**

Note. Loadings > .30 shown, with greatest value per factor in bold.

B4 *Descriptive Statistics Including Means and Standard Deviations for EST-Q Mean Subscales by Type of Therapist and Subscale Total Score.*

	Therapist-Type	*n*	*Range*	*M* (*SD*)
EMDR Subscale Mean Total	Any	346	1 – 5	3.17 (1.05)
	CBT	166		2.47 (0.56)
	EMDR	30		4.24 (0.55)
	Both	105		4.18 (0.74)
	Neither	22		2.26 (0.70)
CBT Subscale Total	Any	344	1 – 5	3.58 (0.71)
	CBT	166		3.75 (0.64)
	EMDR	30		3.30 (0.60)
	Both	105		3.53 (0.69)
	Neither	22		2.88 (0.81)
Both Subscale Total	Any	341	2 – 5	4.24 (0.53)
	CBT	166		4.24 (0.50)
	EMDR	30		4.19 (0.50)
	Both	105		4.35 (0.43)
	Neither	22		3.94 (0.81)
Exposure Subscale Total	Any	345	1 – 5	2.76 (0.93)
	CBT	166		2.93 (0.97)
	EMDR	30		2.58 (0.83)
	Both	105		2.68 (0.88)
	Neither	22		2.26 (0.87)

Table B5 Within Therapist-Type Contrasts of EST-Q Mean Subscale Scores.

EST-Q Subscale	Therapist Type	Therapist Type	Mean Difference	SE	*p*	Lower Bound	Upper Bound
EMDR Mean	CBT	EMDR	-1.76	.11	.001	-2.05	-1.46
		Both	-1.70	.08	.001	-1.92	-1.48
		Neither	0.25	.15	.34	-0.15	-0.65
	EMDR	CBT	-	-	-	-	-
		Both	0.06	.12	.97	-0.27	0.39
		Neither	2.01	.17	.001	1.55	2.47
	Both	CBT	-	-	-	-	-
		EMDR	-	-	-	-	-
		Neither	1.95	.16	.001	1.53	2.37
CBT Mean	CBT	EMDR	0.46	.12	.003	0.13	0.78
		Both	0.22	.08	.05	0.00	0.43
		Neither	0.91	.18	.001	0.43	1.39
	EMDR	CBT	-	-	-	-	-
		Both	-0.24	.13	.26	-0.58	0.10
		Neither	0.45	.20	.13	-0.09	0.99
	Both	CBT	-	-	-	-	-
		EMDR	-	-		-	-
		Neither	0.69	.18	.004	0.20	1.18
Both Mean	CBT	EMDR	0.04	.10	.97	-0.22	0.31
		Both	-0.12	.06	.15	-0.27	0.03
		Neither	0.34	.18	.23	-0.14	0.82
	EMDR	CBT	-	-	-	-	-
		Both	-0.17	.10	.36	-0.44	0.10
		Neither	0.30	.19	.43	-0.22	0.82
	Both	CBT	-	-	-	-	-
		EMDR	-	-	-	-	-
		Neither	0.46	.18	.06	-0.02	0.95
	CBT	EMDR	0.35	.17	.18	-0.10	0.80

Exposure Mean		Both	0.25	.11	.12	-0.04	0.55
		Neither	0.66	.19	.01	0.14	1.19
	EMDR	CBT	-	-	-	-	-
		Both	-0.10	.17	.94	-0.56	0.37
		Neither	0.31	.23	.55	-0.31	0.94
	Both	EMDR	-	-	-	-	-
		CBT	-	-	-	-	-
		Neither	0.41	.20	.19	-0.13	0.95

Supplement C: Summary of supplemental regression analyses

To provide more information about the relationship between the EST components (EST-Q subscales) and the potential that other therapist factors may account for the relationship between the personality factors, counseling self-efficacy and trait anxiety, regression analyses were used. Each EST-Q subscale (EMDR, CBT, Both, and Exposure) were each entered separately as the dependent variable (for four separate analyses), and personality traits of extraversion, agreeableness, openness to experience, conscientiousness, and neuroticism, as well as self-efficacy subscales, state and trait anxiety, and several other covariates including type of therapist training – (the four types CBT, EMDR, Both, or Neither were dummy coded EMDR versus other, Both versus other and neither versus other), age, gender, years practicing, type of degree – masters or doctoral, and years since completing their terminal degree) were entered simultaneously as independent variables to predict each of the four subscales separately. As shown below, the big five personality indicators were not predictive of any EST subscale but the COSE: Difficult Client Behaviors subscale remained significantly associated with the EST-Q EMDR, CBT and Both Subscales.

Table C1. *Summary of Regression Analyses Predicting EMDR Subscale Score*

	Slope (S.E.)	t	Semi-partial
Years since completing degree	0.01 (0.01)	1.44	.05
Age	-0.00 (0.01)	-0.76	-.03
Gender	-0.15 (0.12)	-3.35	-.12
Type of Degree	-0.30 (0.09)	-3.35^{***}	-.12
COSE: Microskills	0.02 (0.01)	2.10	.07
COSE: Process	-0.01 (0.01)	-1.07	-.04
COSE: Difficult Client Behaviors	0.03 (0.01)	2.66^{**}	.09
COSE: Cultural Competence	0.01 (0.02)	0.39	.01
COSE: Values	0.01 (0.02)	0.70	.02
Trait Anxiety	-0.00 (0.01)	-0.07	-.00
State Anxiety	0.01 (0.01)	1.36	.05
EMDR Trained Therapist	1.79 (0.16)	11.36^{***}	.40
Both Trained Therapist	1.70 (0.09)	18.88^{***}	.66
Neither Trained Therapist	-0.32 (0.16)	-2.04^{*}	-.07
Extraversion	0.01 (0.01)	0.74	.03
Agreeableness	-0.00 (0.01)	-0.32	-.01
Conscientiousness	-0.01 (0.01)	-1.51	-.05
Neuroticism	-0.01 (0.01)	-0.57	-.02
Openness	.00 (.01)	0.28	.01

$^{*}p < .05$. $^{**}p < .01$. $^{***}p < .001$. *Note.* EST-Q = Therapists' Experiences with Empirically Supported Treatments Questionnaire.

Table C2. *Summary of Regression Analyses Predicting CBT Subscale Score*

	Slope (S.E.)	t	Semi-partial
Years since completing degree	-0.01 (0.01)	-2.20	-.13
Age	0.01 (0.01)	1.90	.12
Gender	-0.38 (0.13)	-2.94*	-.18
Type of Degree	-0.07 (0.10)	-0.67	-.04
COSE: Microskills	0.01 (0.01)	1.39	.08
COSE: Process	-0.00 (0.01)	-0.38	-.02
COSE: Difficult Client Behaviors	0.02 (0.01)	1.55**	.09
COSE: Cultural Competence	0.01 (0.02)	0.56	.03
COSE: Values	0.00 (0.02)	0.14	.01
Trait Anxiety	-0.01 (0.01)	-1.04	-.06
State Anxiety	0.01 (0.01)	0.92	.06
EMDR Trained Therapist	-0.35 (0.17)	-2.00	-.12
Both Trained Therapist	-0.22 (0.10)	-2.25	-.14
Neither Trained Therapist	-0.89 (0.18)	-4.91**	-.30
Extraversion	0.01 (0.01)	0.80	.05
Agreeableness	-0.01 (0.01)	-0.57	-.03
Conscientiousness	-0.01 (0.01)	-1.21	-.07
Neuroticism	-0.02 (0.01)	-1.61	-.10
Openness	-0.02 (0.01)	-1.61	-.10

*$p < .05$. **$p < .01$. *Note.* EST-Q = Therapists' Experiences with Empirically Supported Treatments Questionnaire.

Table C3. *Summary of Regression Analyses Predicting Both Subscale Score*

	Slope (S.E.)	*t*	Semi-partial
Years since completing degree	0.01 (0.01)	1.18	.07
Age	-0.01 (0.01)	-1.35	-.08
Gender	-0.25 (0.10)	-2.48**	-.15
Type of Degree	-0.10 (0.08)	-1.37	-.08
COSE: Microskills	-0.00 (0.01)	-0.36	-.08
COSE: Process	-0.01 (0.01)	-1.21	-.07
COSE: Difficult Client Behaviors	0.02 (0.01)	2.76**	.17
COSE: Cultural Competence	0.02 (0.01)	1.48	.09
COSE: Values	0.01 (0.01)	0.91	.06
Trait Anxiety	-0.00 (0.01)	-0.42	-.03
State Anxiety	0.00 (0.01)	0.21	.01
EMDR Trained Therapist	-0.08 (0.14)	-0.60	-.04
Both Trained Therapist	0.13 (0.01)	1.63	.10
Neither Trained Therapist	-0.44 (0.14)	-3.20**	-.20
Extraversion	0.00 (0.01)	0.38	.02
Agreeableness	0.01 (0.01)	0.64	.04
Conscientiousness	-0.01 (0.01)	-0.91	-.06
Neuroticism	0.00 (0.01)	0.02	.00
Openness	-0.00 (0.01)	-0.28	-.01

**$p < .01$. *Note.* EST-Q = Therapists' Experiences with Empirically Supported Treatments Questionnaire

Table C4. *Summary of Regression Analyses Predicting Exposure Subscale Score*

	Slope (S.E.)	*t*	Semi-partial
Years since completing degree	0.01 (0.01)	0.64	.04
Age	-0.01 (0.01)	-1.35	-.08
Gender	-0.28 (0.18)	-1.52	-.10
Type of Degree	0.19 (0.14)	1.38	.09
COSE: Microskills	0.01 (0.01)	0.53	.04
COSE: Process	0.00 (0.01)	0.37	.02
COSE: Difficult Client Behaviors	0.03 (0.02)	1.78	.12
COSE: Cultural Competence	0.01 (0.02)	0.36	.02
COSE: Values	-0.01 (0.03)	-0.22	-.01
Trait Anxiety	0.00 (0.02)	0.25	.02
State Anxiety	0.00 (0.01)	0.39	.03
EMDR Trained Therapist	-0.12 (0.25)	-0.49	-.03
Both Trained Therapist	-0.00 (0.14)	-0.03	-.00
Neither Trained Therapist	-0.39 (0.26)	-1.51	-.10
Extraversion	-0.00 (0.01)	-0.33	-.02
Agreeableness	-0.03 (0.02)	-1.42	-.09
Conscientiousness	-0.03 (0.01)	-1.77	-.12
Neuroticism	-0.02 (0.02)	-1.19	-.08
Openness	-0.01 (0.01)	-0.89	-.06

Note. EST-Q = Therapists’ Experiences with Empirically Supported Treatments Questionnaire
